# Toxicokinetic Characterization of MDM Hydantoin via Stable Metabolite DMH: Population Modeling for Predicting Dermal Formaldehyde Formation

**DOI:** 10.3390/toxics13110917

**Published:** 2025-10-25

**Authors:** Woohyung Jung, Jaewoong Lee, Woojin Kim, Seongwon Kim, Woojin Nam, In-Soo Myeong, Kwang Ho Kim, Soyoung Shin, Tae Hwan Kim

**Affiliations:** 1 College of Pharmacy, Daegu Catholic University, Gyeongsan 38430, Republic of Korea; 2 Convergence Research Institute for Biomedical Sciences, Daegu Catholic University, Gyeongsan 38430, Republic of Korea; 3 Gyeongbuk Technopark Medical Convergence Materials Commercialization Center, Gyeongsan 38430, Republic of Korea; 4 College of Pharmacy, Chung-Ang University, Seoul 06974, Republic of Korea

**Keywords:** MDMH, DMH, toxicokinetics, population toxicokinetic modeling, formaldehyde releaser

## Abstract

MDM hydantoin (MDMH), a formaldehyde-releasing preservative widely used in cosmetics, poses potential health risks due to its conversion to formaldehyde and systemically absorbed metabolites. Current safety assessments lack quantitative exposure data due to rapid degradation of MDMH in biological matrices. In the present study, we developed a validated LC-MS/MS assay for simultaneous determination of MDMH and its stable metabolite DMH in rat plasma, and characterized their toxicokinetics using population modeling following intravenous and transdermal administration. MDMH exhibited extremely rapid elimination (t_1/2_ = 0.4 ± 0.1 min) with near-complete conversion to DMH (97.6 ± 9.6%), while DMH demonstrated prolonged retention (t_1/2_ = 174.2 ± 12.2 min) and complete bioavailability (100.9 ± 18.0%) after transdermal application. Population modeling estimated that 84% (relative standard error: 42.8%) of applied MDMH undergoes cutaneous absorption and metabolism to DMH and formaldehyde within skin tissues. This study demonstrates that stable metabolite monitoring combined with population modeling enables toxicokinetic characterization of rapidly degrading compounds following dermal exposure.

## 1. Introduction

1-(Hydroxymethyl)-5,5-dimethylhydantoin (MDM hydantoin, MDMH), one of the commonly used formaldehyde-releasing preservatives in the cosmetic industry, serves as an effective antimicrobial agent [1]. The molecular formulas of MDMH and its major metabolite 5,5-dimethylhydantoin (DMH) are C_6_H_10_N_2_O_3_ and C_5_H_8_N_2_O_2_, respectively.

**Figure 1:**
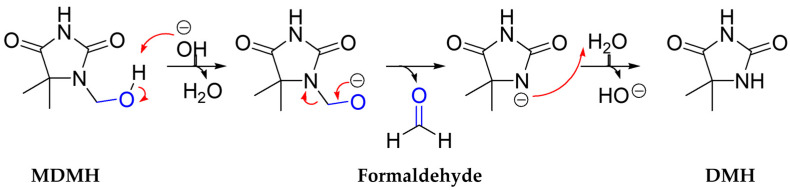
Chemical conversion of MDMH to DMH and formaldehyde.

The mechanism of formaldehyde release from MDMH involves hydrolytic cleavage of the N-hydroxymethyl group, resulting in the liberation of one equivalent of formaldehyde and the formation of DMH as the primary metabolite (Figure 1) [1,2]. Similarly to other formaldehyde-releasing preservatives, this decomposition process is likely influenced by environmental factors such as pH, temperature, and matrix composition [1]. These preservatives are expected to be degraded in biological environments. Thus, when MDMH-containing products are applied to skin, both the parent compound and released formaldehyde may be exposed to skin and systemic circulation, potentially causing various toxic effects.

Current regulations permit MDMH concentrations up to 0.2% in cosmetic products [3,4]. However, these concentration limits were established without quantitative approach on systemic absorption and metabolite disposition patterns. This limitation in exposure assessment is particularly concerning given that formaldehyde-releasing preservatives are designed to continuously generate the active preservative throughout product shelf-life and consumer use.

The most frequently reported adverse effects associated with MDMH include allergic reactions caused by skin contact with released formaldehyde [5,6,7]. Previous studies have demonstrated that increased usage of MDMH in cosmetics has elevated the risk of cosmetic dermatitis in users with formaldehyde sensitivities [8]. In addition to localized effects, systemic exposure to formaldehyde and its metabolites raises additional safety concerns that require quantitative characterization for risk assessment.

Toxicokinetic evaluation of MDMH involves analytical challenges due to the instability of the parent compound in biological matrices. While several analytical methods exist for determining MDMH and related compounds in cosmetic products using HPLC [9,10], analytical methods for MDMH in biological matrices have not been reported. The lack of bioanalytical methods has resulted in limited quantitative data regarding local and systemic exposure of MDMH and formaldehyde following transdermal application.

DMH, the primary metabolite lacking the chemically reactive N-hydroxymethyl group, exhibits greater stability in biological matrices than MDMH. This stability suggests that DMH may serve as a surrogate marker for assessing internal exposure of MDMH. Characterizing toxicokinetics of DMH following both MDMH and DMH provides quantifiable information on the absorption and metabolic properties of the parent compound, which is difficult to be directly determined due to its rapid degradation property in biological samples.

Accurate characterization of toxicokinetic properties is essential for comprehensive risk assessment of toxicants. Population toxicokinetic modeling integrates and analyzes the absorption and distribution properties of parent and metabolite compounds administered via various routes, enabling the characterization of toxicokinetic disposition for substances whose internal exposure is difficult to observe. This approach accommodates interindividual variation in response, facilitating human exposure and risk assessment [11]. Current risk assessment approaches have primarily relied on in vitro measurements of formaldehyde concentrations in product formulations under controlled conditions [1], combined with modeling-based exposure calculations [12,13]. These assessments have derived dermal absorption parameters from literature values or animal studies rather than direct measurement in relevant exposure scenarios [13]. While these quantitative approaches have advanced the field, the evaluation of systemic exposure [12], remains challenging without in vivo toxicokinetic data characterizing actual absorption rates, metabolic conversion fraction.

Overall, this study aimed to establish a toxicokinetic characterization approach for analytically challenging compounds using stable metabolite analysis and population modeling, with MDMH as a model compound.

## 2. Materials and Methods

### 2.1. Materials and Reagents

1-(Hydroxymethyl)-5,5-dimethyl hydantoin (MDM hydantoin; MDMH) (purity 98%) was purchased from Tokyo Chemical Industry Co., Ltd. (Tokyo, Japan). 5,5-Dimethyl hydantoin (DMH; DMH) (purity 97%) and acrylamide (purity 99%, internal standard, IS) were purchased from Sigma-Aldrich Chemical Co., Ltd. (St. Louis, MO, USA). Methanol, distilled water [high-performance liquid chromatography (HPLC) grades] were purchased from J. T. Baker, Inc. (Phillipsburg, NJ, USA). HPLC grade formic acid and hydrochloric acid were purchased from Merck (Union, NJ, USA).

### 2.2. Analytical Assay

#### 2.2.1. Instruments

An API 4000 LC-MS/MS system (Applied Biosystems/MDS Sciex, Foster City, CA, USA) coupled with an Agilent 1100 HPLC (Agilent Technologies, Santa Clara, CA, USA) was used for determination of MDMH and DMH in rat plasma. The analytes were separated on a Synergi Fusion-RP 80 Å (150 × 2.0 mm, i.d., 4 µm;) with a Security Guard™ Cartridge AJ0-7556 (Phenomenex, Torrance, CA, USA), and the column oven temperature was maintained at 30 °C. An isocratic solvent system of distilled water and methanol (80:20, *v*/*v*) was used with a runtime of 7 min. The injection volume was 10 µL, and the flow rate was maintained at 0.2 mL/min.

To detect the MDMH and DMH applying mass spectrometry, an ion spray interface with positive ion multi-reaction monitoring (MRM) mode was used with a dwell time of 200 ms. The MRM transitions were set at 159.0 → 84.0 *m*/*z* for MDMH, 129.0 → 58.0 *m*/*z* for DMH, and 72.0 → 55.0 *m*/*z* for acrylamide (IS). The ion spray voltage was 5500 V, and the turbo gas temperature was 450 °C. The ion source gas 1, 2, and entrance potential were optimized, 50, 55 L/h, and 10 V, respectively. The declustering potential was set at 41, 51, and 41 V for MDMH, DMH, and IS, respectively. The collision energy was set at 31, 31, and 19 V for MDMH, DMH, and IS, respectively. Data acquisition was carried out with Analyst 1.6.3 (Applied Biosystems/MDS Sciex, Foster City, CA, USA).

#### 2.2.2. Stock and Working Solutions

Individual stock solutions (1 mg/mL) of MDMH and DMH were prepared by dissolving 10 mg of MDMH and DMH in 10 mL of methanol containing 5% formic acid and 0.1 M HCl. Stock solution of IS was prepared with 0.5% formic acid in methanol at a concentration of 1 mg/mL.

The working solutions of MDMH and DMH were mixed and diluted with methanol containing 5% formic acid and 0.1M HCl at concentrations of 0.1, 0.2, 0.5, 1, 2, 5, and 10 µg/mL. IS was diluted with methanol containing 0.5% formic acid at a concentration of 1 µg/mL. All stock and working solutions were stored in amber glass vials at −70 °C to prevent potential photodegradation and thermal decomposition until analysis.

#### 2.2.3. Preparation of Calibration Standard and Quality Control Samples

Calibration standard samples were prepared by spiking 50 µL of working solutions into 50 µL of rat plasma containing 0.5% formic acid to achieve final concentrations of 0.1, 0.2, 0.5, 1, 2, 5, and 10 µg/mL. Quality control (QC) samples were prepared at concentrations of 0.1 (Lower limit of quantification), 0.3 (Low QC), 4 (Medium QC), and 8 (High QC) µg/mL using the same method. All calibration standards and QC samples were stored at −70 °C until analysis.

#### 2.2.4. Sample Preparation

For plasma sample preparation, formic acid was immediately added to blood samples upon collection to achieve a final concentration of 0.5% to prevent potential enzymatic degradation of MDMH, and all sample preparation procedures were conducted at ≤4 °C to minimize temperature-dependent decomposition of the analytes. For analysis, 50 μL of internal standard solution and 100 μL of methanol containing 0.5% formic acid (as precipitating solvent) were added to 50 μL of plasma, followed by vortex-mixing for 2 min. After centrifugation at 21,206× *g* for 10 min, 40 μL of supernatant was mixed with 160 μL of 0.5% formic acid in distilled water, and 10 μL was injected into the LC-MS/MS system.

#### 2.2.5. Method Validation

Intra- and inter-day accuracy and precision were assessed by analyzing three replicates of lower limit of quantification (LLOQ) and quality control (low, medium, and high) samples over three consecutive days. Accuracy was determined as the percentage difference between measured concentrations and nominal concentrations. Precision was expressed as the coefficient of variation for each concentration level. Both accuracy and precision met acceptance criteria of ±15% for all concentrations, except LLOQ which was within ±20%.

Selectivity was assessed by analyzing blank plasma and blank plasma spiked with MDMH, DMH, and internal standard. Calibration curves were constructed using weighted linear regression (1/x) of the peak area ratios of analytes to internal standard versus nominal concentrations. Correlation coefficients exceeded 0.999 for all analytes. The LLOQ was defined as the lowest calibration standard concentration that demonstrated signal-to-noise ratios ≥10 with acceptable accuracy and precision (≤20%).

Stability studies were conducted using four replicates of low and high quality control samples under various conditions. Short-term stability of MDMH and DMH in rat plasma was evaluated at room temperature for 4 h. Long-term stability was assessed following storage at −20 °C for 4 weeks. Freeze–thaw stability was determined after three freeze–thaw cycles. Additionally, processed sample stability was evaluated in the autosampler at 4 °C for 24 h. Stability was considered acceptable when the mean deviation from theoretical concentrations was within ±15%.

### 2.3. Animal Studies

#### 2.3.1. Animals

All animal care and experiments were conducted to evaluate the toxicokinetics of MDMH and DMH according to the Guidelines for the Care and Use of Animal approved by the Ethics Committee for Treatment of Laboratory Animal at Daegu Catholic University (CUD-2019-020). To evaluate the toxicokinetics of MDMH and DMH in rats after intravenous injection (IV) or transdermal application (TD). Male Sprague-Dawley rats (9 weeks, body weight 260–310 g) (Hyochang Science Co., Daegu, Republic of Korea) were kept in plastic cages and fed regularly with water and feed. Rats were acclimated for a minimum of 1 week prior to the experiment under conditions keeping a 12 h light-dark cycle, a temperature of 23 ± 2 °C, and a relative humidity of 50 ± 10%. The rats were cannulated using polyethylene tubing (0.58 mm i.d., 0.96 mm o.d., Natsume, Tokyo, Japan) in the jugular vein after intraperitoneal injection at a dose of 0.9 mL/kg of Zoletil 50 (Virbac, Carros, France).

#### 2.3.2. Intravenous Injection Study

For IV injection, MDMH or DMH was dissolved in distilled water, respectively, and the dose was 50 or 10 mg/kg, respectively. Blood samples were collected via the jugular vein in the MDMH administration group at 0, 0.5, 1, 1.5, 2, 2.5, 3, 5, 10, and 15 min and 1, 2, 4, 8, 12, and 24 h (16 points/head), and in the DMH administration group at 0, 2, 5, 10, 15, 30 min and 1, 2, 4, 8, 12, and 24 h (12 points/head). Plasma samples were harvested by centrifugation of obtained blood for 10 min at 4000× *g*, and stored at −20 °C until analysis.

#### 2.3.3. Transdermal Application Study

The transdermal application study was performed according to the “Organization for Economic Cooperation and Development guidelines for the in vivo skin absorption test”. Rats were anesthetized using inhaled isoflurane, and the back area of 6 × 6 cm^2^ was removed using an electric clipper (808, Daito Electric Co., Osaka, Japan) 12 h before administration. The clipped skin of the rat was wiped with acetone without damaging the stratum corneum, and a resting period was allowed just before the study.

The compositions of gel containing MDMH and DMH are shown in Table 1. For TD application, a gel corresponding to approximately 5 mg/cm^2^ was applied to an area of 5 × 5 cm^2^ among the depilated areas. The doses of MDMH and DMH for the transdermal application study were 10.9 and 10.7 mg/kg on average, respectively. Although the regulatory limit for MDMH in cosmetics is 0.2%, a higher concentration (2%) was employed to ensure adequate systemic exposure and plasma concentrations suitable for accurate toxicokinetic characterization of these rapidly metabolized compounds. To prevent loss of the compounds due to volatilization and grooming, the application on the back site was adhered with gauze and pads. Blood samples were collected via the jugular vein in the MDMH application group at 0, 30 min, and 1, 2, 3, 4, 5, 6, 7, 8, 10, 12, and 24 h (13 points/head), and in the DMH application group at 0, 30 min, and 1, 2, 3, 4, 6, 8, 10, 12, and 24 h (11 points/head). Plasma samples were harvested by centrifugation of obtained blood for 10 min at 4000× *g*, and stored at −20 °C until analysis.

### 2.4. Noncompartmental Analysis

The toxicokinetic parameters were calculated by standard non-compartmental analysis using the non-linear least squares regression program Phoenix™ WinNonlin^®^ 6.1 (Pharsight, Cary, NC, USA).

The non-compartmental toxicokinetic parameters included terminal half-life (t_1/2_), area under the plasma concentration versus time curve from time zero to the last observation time point (AUC_last_) and to infinity (AUC_infinity_), volume of distribution (V_z_), and systemic clearance (CL). The maximum plasma concentration (C_max_) and the time to reach C_max_ (T_max_) were obtained directly from the observed data. Absolute bioavailability (F) was estimated as the ratio of the dose-normalized AUC_infinity_ of MDMH and DMH following TD application compared to IV injection. The fraction metabolized (F_m_) was calculated as the ratio of metabolite AUC to parent AUC after correction for molecular weight differences, accounting for the stoichiometric conversion relationship:

(1) $$F_{m}=\frac{{AUC}_{DMH}\cdot {MW}_{MDMH}}{{AUC}_{MDMH}\cdot {MW}_{DMH}}$$

where MWMDMH = 158.16 g/mol and MWDMH = 128.13 g/mol. This parameter represents the proportion of administered MDMH that is converted to DMH in vivo, reflecting the efficiency of the metabolic conversion process. Following transdermal application, F_m_ was calculated as the ratio of DMH exposure after MDMH gel application to that after direct DMH gel application, representing the combined efficiency of MDMH transdermal absorption, metabolic conversion to DMH, and subsequent systemic absorption of the formed metabolite.

### 2.5. Population Toxicokinetic Modeling

POP-TK modeling was performed to develop a compartment model describing the disposition of MDMH and DMH following different administration routes. Plasma concentration data from all studies were combined for model development.

The compartment model was designed to fit the absorption phase following TD application, disposition kinetics of MDMH and DMH following IV injection. The disposition phase of MDMH was described with a one-compartment model. Following TD application of MDMH, only DMH was detected in plasma, while MDMH concentrations remained below the quantification limit due to its rapid elimination in the systemic circulation.

For transdermal application, MDMH applied to the skin undergoes simultaneous absorption and metabolic conversion to DMH and formaldehyde. This process was described by a first-order rate constant (k_a,met_) representing the combined absorption-metabolism kinetics. The parameter F_a,met_ represents the fraction of applied dose that penetrates the skin and undergoes metabolic conversion, accounting for incomplete absorption.

The equation for MDMH amount in the administration site (X_Skin,MDMH_) was

(2) $$\frac{dX_{Skin,MDMH}}{dt}=-k_{m}\cdot X_{Skin,MDMH}$$

DMH formed in the skin compartment subsequently undergoes absorption into systemic circulation. This absorption was modeled using a first-order rate constant (k_a,DMH_) with three transit compartments to describe the delayed appearance of DMH in plasma.

The equations for DMH in the skin depot and transit compartments were:

(3) $$\frac{dX_{Skin,DMH}}{dt}=-k_{a,DMH}\cdot X_{Skin,DMH}+F_{a,met}\cdot (\frac{{MW}_{DMH}}{{MW}_{MDMH}}){\cdot k}_{a,met}\cdot X_{Skin,MDMH}$$

(4) $$\frac{dX_{Transit1,DMH}}{dt}=+k_{a,DMH}\cdot X_{Skin,DMH}-k_{a,DMH}\cdot X_{Transit1,DMH}$$

(5) $$\frac{dX_{Transit2,DMH}}{dt}=+k_{a,DMH}\cdot X_{Transit1,DMH}-k_{a,DMH}\cdot X_{Transit2,DMH}$$

(6) $$\frac{dX_{Transit3,DMH}}{dt}=+k_{a,DMH}\cdot X_{Transit2,DMH}-k_{a,DMH}\cdot X_{Transit3,DMH}$$

The disposition of MDMH and DMH was assessed using a linear model with a central and one, two, or three peripheral compartments. The model incorporated both skin metabolism and systemic metabolism of MDMH to DMH. Systemic metabolism was described by the systemic clearance, representing the fraction of eliminated MDMH that is converted to DMH in the central compartment. Molecular weight correction was applied to account for stoichiometric conversion relationships.

The final disposition and elimination compartment model was determined to use one central compartment for MDMH and one central and one peripheral compartment for DMH based on the objective function.

The differential equations for the amounts of MDMH in the central (X_Central,MDMH_) compartments and DMH in the central (X_Central,DMH_) and peripheral (X_Peripheral,DMH_) compartments were:

(7) $$\frac{dX_{Central,MDMH}}{dt}=-{CL}_{MDMH}\cdot C_{Central,MDMH}$$

(8) $$\frac{dX_{Central,DMH}}{dt}=-{CL}_{DMH}\cdot C_{Central,DMH}-{CLD}_{DMH}\cdot C_{Central,DMH}+{CLD}_{DMH}\cdot \;C_{Peripheral,DMH}+k_{a,DMH}\cdot X_{Transit3,DMH}+(\frac{{MW}_{DMH}}{{MW}_{MDMH}})\cdot {CL}_{MDMH}\cdot C_{Central,MDMH}$$

(9) $$\frac{dX_{Peripheral,DMH}}{dt}=+{CLD}_{DMH}\cdot C_{Central,DMH}-{CLD}_{DMH}\cdot C_{Peripheral,DMH}$$

The formaldehyde compartment was designed to predict formaldehyde formation from MDMH metabolism using appropriate molecular weight correction factor. Formaldehyde formation was assumed to occur simultaneously with MDMH in the skin compartment with a 1:1 stoichiometric ratio.

The equation of release kinetics to formaldehyde from MDMH in the Skin (X_Skin,Formaldehyde_) compartment was:

(10) $$\frac{dX_{Skin,Formaldehyde}}{dt}=+F_{a,met}\cdot (\frac{{MW}_{Formaldehyde}}{{MW}_{MDMH}})\cdot k_{a,met}\cdot X_{Skin,MDMH}$$

C_Central,MDMH_ and C_Central,DMH_ represent MDMH and DMH plasma concentrations in respective compartments and C_Peripheral,DMH_ represent DMH concentration in peripheral compartment. CL_MDMH_ and CL_DMH_ were systemic clearance of MDMH and DMH and CLD_DMH_ was distribution clearance.

The plasma concentrations of MDMH and DMH were simultaneously modeled and predicted using POP-TK modeling with the stochastic approximation expectation maximization (SAEM) algorithm in the parallelized Monolix^®^ 2023R1 (Lixoft, Antony, Paris, France) and Berkeley Madonna version 8.3.11 (University of California at Berkeley, Berkeley, CA, USA). An importance sampling SAEM was used for population parameter estimation to describe the between-subject variability (BSV) for each parameter estimate. The final model was determined based on goodness-of-fit, visual inspection of diagnostic plots, and physiological plausibility, which collectively suggested this structure provided the most robust and parsimonious fit to the observed data.

## 3. Results

### 3.1. Mass Spectrometry and Chromatography

**Figure 2:**
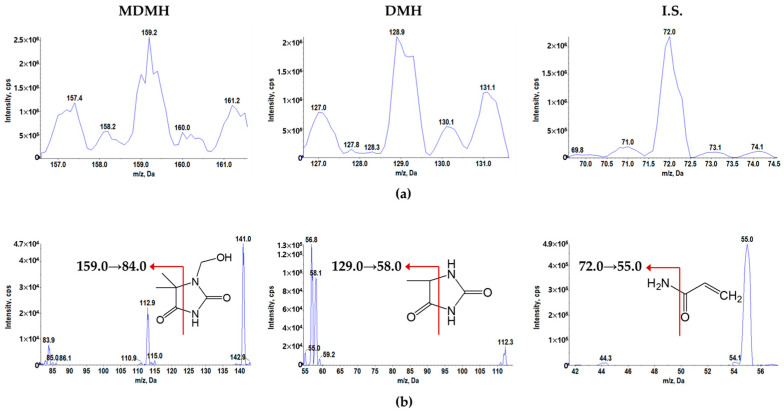
(**a**) Q1 and (**b**) product ion mass spectra of protonated MDMH, DMH, and I.S. in positive ionization mode.

The MRM mass transitions for quantitative analysis were determined by evaluating the sensitivity of protonated ions and product ion spectra. The Q1 and product ion mass spectra of protonated MDMH, DMH, and internal standard are shown in Figure 2. The most abundant ions in the full scan Q1 mass spectrum were the protonated molecular ions of MDMH, DMH, and acrylamide (internal standard), observed at *m*/*z* 159.0, 129.0, and 72.0, respectively. In the product ion scan mass spectra, the most prominent product ions were observed at *m*/*z* 84.0 for MDMH, 58.0 for DMH, and 55.0 for acrylamide. Subsequently, the MRM transitions were set at 159.0 → 84.0 *m*/*z* for MDMH, 129.0 → 58.0 *m*/*z* for DMH, and 72.0 → 55.0 *m*/*z* for acrylamide (IS).

To prepare plasma samples, simple protein precipitation was employed. Chromatographic separation was achieved using a Synergi Fusion-RP column with an isocratic mobile phase of distilled water and methanol (80:20, *v*/*v*). The retention times for MDMH, DMH, and internal standard were 3.99, 3.89, and 2.80 min, respectively, with no interfering peaks observed that would affect quantification.

**Figure 3:**
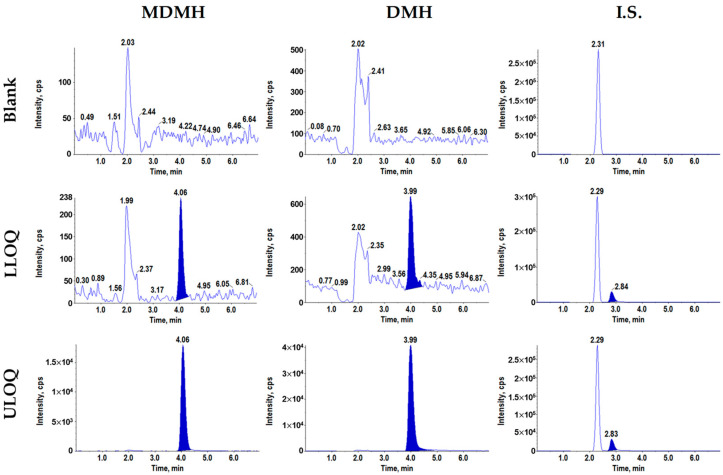
MRM chromatograms of MDMH, DMH, and acrylamide (IS) obtained by extraction of blank plasma (**upper panel**), LLOQ concentration spiked in plasma (**middle panel**, 0.1 μg/mL), and ULOQ concentration spiked in plasma (**lower panel**, 10 μg/mL).

Figure 3 presents representative MRM chromatograms obtained from blank plasma, LLOQ concentration spiked plasma, and upper limit of quantification (ULOQ) concentration spiked plasma. The chromatograms demonstrated adequate sensitivity and selectivity for both analytes across the analytical range. The calibration curves were linear over concentration ranges of 0.2–100 μg/mL for MDMH and 0.5–100 μg/mL for DMH with correlation coefficients (r^2^) greater than 0.999 for both analytes.

### 3.2. Assay Accuracy, Precision, and Stability

Intra- and inter-day accuracy and precision were evaluated using quality control samples at four concentration levels (LLOQ, low, medium, and high). As summarized in Table 2, intra-day accuracy ranged from 102.8% to 111.4% for MDMH and 100.8% to 104.3% for DMH. Intra-day precision was ≤5.2% for MDMH and ≤9.1% for DMH. Inter-day accuracy and precision values were within the acceptable range of ±15% for both analytes (±20% for LLOQ), meeting the acceptance criteria specified in regulatory guidelines.

Stability studies were conducted using five replicates of low and high quality control samples under various analytical conditions, with results shown in Table 3. Short-term stability was evaluated at room temperature (25 °C) for 4 h, showing mean recoveries within acceptable limits for both analytes. Long-term stability was assessed following storage at −20 °C for 4 weeks, with recoveries remaining within ±15% of theoretical concentrations. Freeze–thaw stability was evaluated after three freeze–thaw cycles. Processed sample stability in the autosampler at 4 °C for 24 h was also within acceptable ranges.

### 3.3. Toxicokinetics of MDMH and DMH in Rats

**Figure 4:**
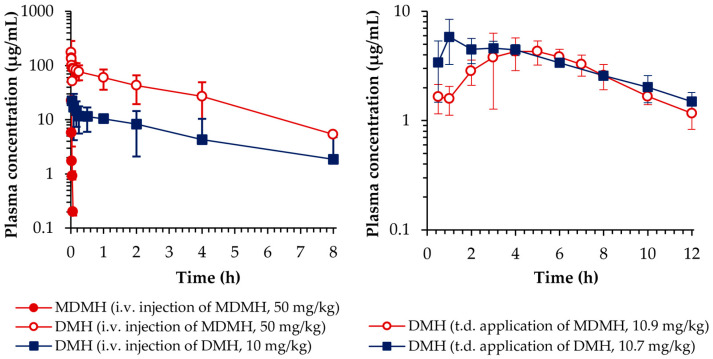
Average plasma concentration-time profiles of MDMH and DMH obtained after IV injection of MDMH at a dose of 50 mg/kg, DMH at a dose of 10 mg/kg, TD application of MDMH at a dose of 10.9 mg/kg, and DMH at a dose of 10.7 mg/kg in rats (n = 5, each).

Following i.v. injection of MDMH, parent compound concentrations reached below the quantification limit within 5 min (Figure 4), while DMH appeared immediately in plasma with peak concentrations reached within 1 min post-injection. This rapid disappearance of MDMH accompanied by immediate DMH appearance indicates rapid and extensive metabolic conversion. Direct intravenous administration of DMH showed a biphasic disposition profile with an initial distribution phase (alpha phase) lasting approximately 30 min, followed by a consistent elimination phase (beta phase).

Table 4 presents the calculated non-compartmental toxicokinetic parameters following IV administration of both compounds. The terminal elimination half-life was 0.4 ± 0.1 min for MDMH and 181.2 ± 33 min for DMH following direct administration. Notably, the elimination half-life of DMH observed after MDMH injection (174.22 ± 12.16 min) was comparable to that following direct DMH administration, indicating that the metabolite exhibits consistent elimination characteristics regardless of its formation pathway. This similarity confirms that the DMH disposition kinetics are not affected by the metabolic conversion process, supporting the validity of using DMH as a biomarker for MDMH exposure assessment.

The metabolic conversion of MDMH to DMH was quantified as 97.59 ± 9.64% based on the AUC ratio adjusted for molecular weight differences, indicating nearly complete and rapid bioconversion of the parent compound. The whole-body clearance was 94.1 ± 34.84 L/h/kg for MDMH and 0.24 ± 0.13 L/h/kg for DMH, reflecting the differences in excretion properties of each compound. These results demonstrate that systemic MDMH exposure results in extensive and rapid conversion to DMH, with the metabolite being the major circulating form.

Transdermal application revealed distinctly different absorption profiles for the two compounds (Figure 4). Following MDMH gel application, parent compound concentrations remained below quantification limits throughout the sampling period, while DMH concentrations gradually increased to reach maximum levels at 4.67 ± 1.53 h. The time-to-peak for DMH following direct transdermal application was shorter (2.0 ± 1.73 h), suggesting that the delayed appearance of DMH after MDMH application reflects the time required for transdermal absorption and metabolic conversion of the parent compound.

Table 5 summarizes the non-compartmental toxicokinetic parameters of DMH following transdermal application of MDMH and DMH gel formulations. The fraction metabolized (Fm) of MDMH, calculated as the ratio of DMH exposure following MDMH application to that following DMH administration, was 89.78 ± 24.15%. This parameter represents the combined efficiency of MDMH transdermal absorption, metabolic conversion to DMH, and subsequent DMH absorption into systemic circulation. Direct DMH application resulted in an absolute bioavailability of 100.92 ± 17.99%. These indicate significant systemic exposure to DMH following transdermal application of both compounds, indicating substantial absorption across the skin barrier.

### 3.4. Population Toxicokinetic Modeling

**Figure 5:**
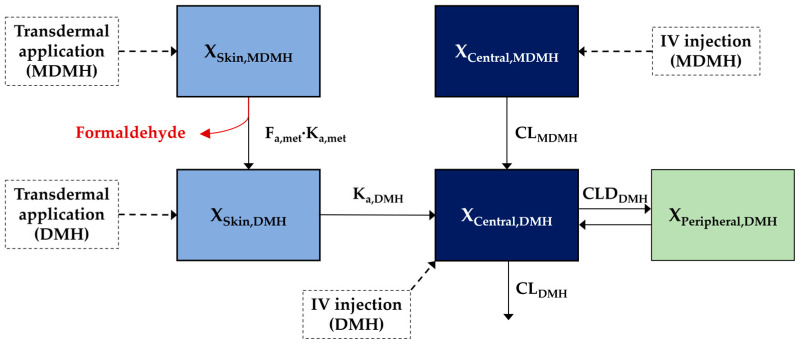
Population toxicokinetic model structure for MDMH and DMH disposition including skin absorption and metabolic conversion pathways.

A comprehensive population toxicokinetic model was developed to simultaneously describe the disposition of both MDMH and DMH across all administration routes and to predict formaldehyde formation patterns. The final population toxicokinetic model structure is depicted in Figure 5, incorporating one-compartment kinetics for MDMH and two-compartment kinetics for DMH, with explicit representation of the metabolic conversion process. Based on the non-compartmental analysis results showing minimal systemic absorption of parent compound following transdermal application, direct absorption of MDMH from skin to systemic circulation was not included in the final model structure.

The model successfully captured the observed disposition profiles, with transdermal MDMH application resulting in gradual DMH appearance reflecting the sequential processes of cutaneous absorption and metabolic conversion at the application site.

Table 6 presents the estimated population toxicokinetic parameters. MDMH clearance was estimated at 147.63 L/h, consistent with the rapid elimination observed across all animals. DMH exhibited lower clearance (0.19 L/h), reflecting the different elimination characteristics of this metabolite.

F_a,met_ was estimated at 0.84 (RSE: 42.8%), indicating that approximately 84% of applied MDMH penetrates the skin and undergoes metabolic conversion to DMH and formaldehyde. The combined absorption-metabolism rate constant (k_a,met_) was estimated at 0.31 h^−1^. The absorption rate constant for DMH (k_a,DMH_), which describes DMH absorption from the skin depot whether from direct application or formation via MDMH metabolism, was estimated at 8.03 h^−1^. These parameters indicate that the majority of topically applied MDMH penetrates the skin barrier and is metabolized rather than remaining on the surface.

**Figure 6:**
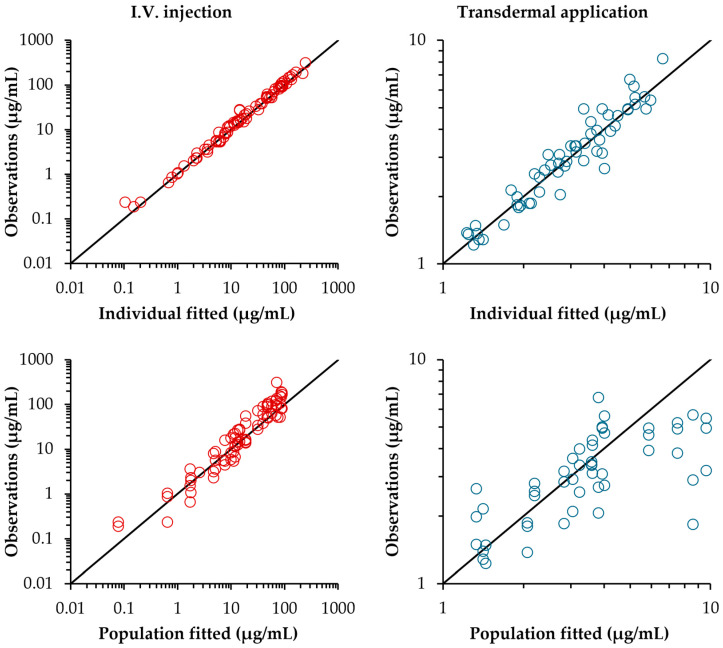
Goodness-of-fit plots for the final population toxicokinetic model showing individual predictions (IPRED, **upper panels**) and population predictions (PRED, **lower panels**) versus observed concentrations (DV) following IV injection (**left**) and TD application (**right**). Solid lines represent the line of identity (slope = 1).

The appropriateness of the model fitting to the observed data was assessed under all dosing conditions. Figure 6 shows a goodness-of-fit plot of the final population model comparing the observed concentrations with individual and group predictions. The diagnostic plots showed acceptable agreement between the observed and predicted concentrations for both individual and group predictions.

**Figure 7:**
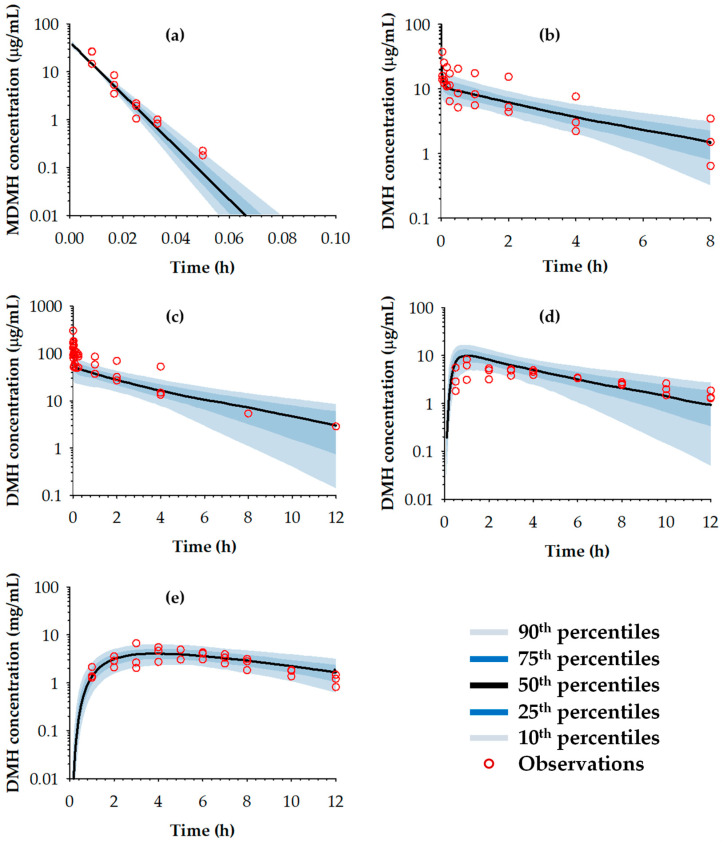
Visual predictive check plots for validation of the final model. (**a**) MDMH concentration following IV injection of MDMH, (**b**) DMH concentration following IV injection of MDMH. (**c**) DMH concentration following IV injection of DMH, (**d**) DMH concentration following TD application of MDMH and (**e**) DMH concentration following TD application of DMH. The lines represented predicted percentiles (10th, 25th, 50th, 75th, 90th) and circles representing the observation.

The visual predictive check plots for model validation are shown in Figure 7. The visual predictive checks showed that observed concentrations fell within the predicted 90% confidence intervals for >90% of data points across all treatment groups, further confirming the adequacy of the final model structure and parameter estimates.

**Figure 8:**
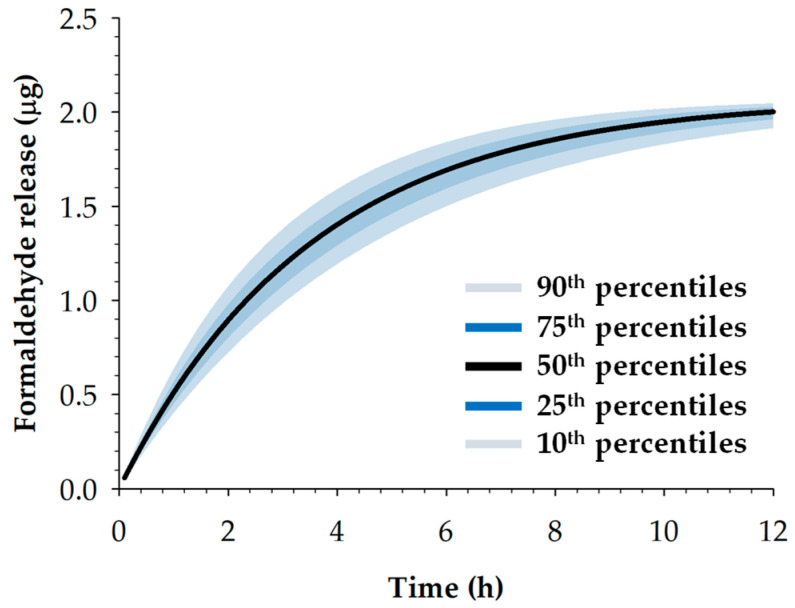
Predicted cumulative formaldehyde formation in skin compartment following transdermal application of 2% MDMH gel (10.9 mg/kg) in rats.

The population toxicokinetic model predicted formaldehyde formation based on the 1:1 stoichiometric conversion of MDMH to DMH and formaldehyde via hydrolytic cleavage. Following transdermal application of 2% MDMH gel (10.9 mg/kg), cumulative formaldehyde formation reached 85% of the theoretical maximum within 8 h, corresponding to degradation of MDMH in the skin (Figure 8).

Using an integrated toxicokinetic experimental and modeling approach, we elucidated the absorption and degradation processes of transdermally administered MDMH, providing quantitative parameters for parent compound elimination and metabolite formation that were previously unavailable due to analytical limitations. The developed model predicted the formation of DMH and formaldehyde in the skin, suggesting that a robust approach has been established to elucidate the internal exposure profile of this highly unstable formaldehyde release preservative.

## 4. Discussion

This study presents toxicokinetic characterization of MDMH and its major metabolite DMH in the safety assessment of formaldehyde-releasing preservatives. The development of a validated LC-MS/MS assay for simultaneous determination of both compounds in biological matrices, combined with population toxicokinetic modeling, provides scientific evidence for regulatory decision-making regarding this cosmetic preservative.

To date, limited analytical methods for MDMH have been reported, and no validated assays for determination of MDMH in biological matrices exist due to its unstable characteristics. Previous analytical approaches were limited to HPLC methods suitable only for cosmetic product analysis [9,10], which are not applicable for characterizing toxicokinetic characteristics including absorption kinetics and metabolism. In the present study, we established a validated LC-MS/MS method for simultaneous determination of both MDMH and its major metabolite DMH in biological matrices through implementation of stabilized conditions (0.5% formic acid, ≤4 °C) during the sample preparation. The analytical method was validated according to FDA and ICH guidelines [14,15]. The instability of MDMH in biological matrices arises from enzymatic degradation of the chemically reactive N-hydroxymethyl group by carboxylesterases, which are abundantly expressed in tissues including skin, liver, and plasma [16,17,18,19]. To address such enzymatic degradation, acidification with formic acid or hydrochloric acid during sample collection and processing has been employed to inhibit esterase activity in biological matrices [20,21].

Using validated analytical methods, we conducted a comprehensive toxicokinetic study of MDMH and estimated the distribution of formaldehyde through a metabolite-based assessment. Both MDMH and DMH were administered intravenously and transdermally, and their toxicokinetic properties were characterized.

The toxicokinetic results demonstrated rapid systemic clearance of MDMH (t_1/2_ = 0.4 ± 0.1 min) and nearly complete conversion to DMH (97.59 ± 9.64%) after intravenous administration. The extensive biotransformation suggests that metabolite-based exposure assessment may provide more reliable data than the unstable parent compound. In other words, the long elimination half-life and high conversion rate of DMH compared to the rapid clearance of MDMH suggest that DMH can be utilized as a biomarker for assessing systemic MDMH exposure. DMH exhibited complete systemic bioavailability (100.92 ± 17.99%) after transdermal application. These results confirm the stability of DMH during skin penetration and suggest that integrated analysis of the collected toxicokinetic data can be used to estimate the distribution of the parent compound and formaldehyde.

To assess exposure to rapidly metabolized compounds, the estimation of parent-to-metabolite conversion ratio is required. In this study, both the parent compound and metabolites were assessed via intravenous and transdermal administration routes, and separate dose studies were conducted for MDMH and DMH. To characterize toxicokinetic properties, the MDMH-to-DMH conversion ratio (97.59 ± 9.64%) was measured. This relationship enables calculation of parent compound exposure from metabolite measurements.

The toxicokinetic findings have important implications for safety assessment of MDMH. The rapid systemic clearance of MDMH (t_1/2_ = 0.4 ± 0.1 min) coupled with near-complete conversion to DMH (97.6 ± 9.6%) indicates that systemic exposure to the parent compound is minimal and transient. This extensive first-pass metabolism suggests that toxicological risk assessment should focus primarily on the metabolite DMH and locally generated formaldehyde, rather than on systemic exposure to MDMH itself. The prolonged retention of DMH (t_1/2_ = 174.2 ± 12.2 min) indicates sustained systemic exposure to the metabolite, warranting consideration in safety assessments of MDMH-containing products.

Population toxicokinetic modeling allowed us to characterize the kinetics of absorption and metabolic transformation. The model indicated that 84% of percutaneously administered MDMH undergoes a combined process of dermal absorption and subsequent metabolism to DMH and formaldehyde within the skin tissue. This suggests that the majority of administered MDMH is absorbed and metabolized, rather than remaining unabsorbed. This integrated modeling approach enables simultaneous assessment of MDMH and metabolite kinetics across multiple administration routes. The population-based analysis provides quantitative insights into the exposure profiles of MDMH, DMH, and formaldehyde that could not be obtained through conventional experimental approaches due to the analytical limitations associated with MDMH instability.

The population modeling approach in this study estimated that approximately 84% of topically applied MDMH may undergo dermal absorption with metabolic conversion within skin tissues. While we did not directly measure formaldehyde concentrations in skin, the stoichiometric relationship between MDMH metabolism and formaldehyde generation suggests potential local formaldehyde exposure during the absorption process. This localized formaldehyde formation may be relevant to understanding the allergenic potential of MDMH-containing cosmetics, as previous clinical studies have documented allergic contact dermatitis in sensitized individuals exposed to formaldehyde-releasing preservatives [5,6,7,8].

Similar dermal absorption kinetics characterization has been applied to risk assessment of other substances with adverse effects. Previous studies have demonstrated that toxicokinetic modeling incorporating dermal absorption kinetics was successfully utilized for exposure and risk assessment of environmental contaminants such as diethyl phthalate and bisphenol A [22,23].

From a systemic perspective, formaldehyde released in skin tissues is expected to undergo rapid enzymatic metabolism to formic acid through well-characterized pathways involving alcohol dehydrogenase and aldehyde dehydrogenase. This metabolic capacity would likely minimize systemic exposure to free formaldehyde. However, our study was conducted in rats, and species differences in skin metabolism and barrier function should be considered when extrapolating these findings to human exposure scenarios. Additionally, while this single-dose study provides insights into acute exposure kinetics, the implications of repeated and prolonged dermal exposure warrant further investigation.

This study provides quantitative data on MDMH absorption and metabolism following dermal application. The estimated fraction undergoing skin penetration and metabolism and the prolonged systemic retention of DMH may contribute to more comprehensive exposure assessments for cosmetic products containing MDMH. While current regulatory limits were established based on available safety data, quantitative toxicokinetic information on dermal absorption and metabolite formation could support future refinements of exposure assessments. Although the present study was conducted in rats, the approach of characterizing exposure through stable metabolite analysis could potentially be adapted for human biomonitoring studies.

The approach of combining stable metabolite analysis with population modeling developed in the present study may have applicability to other formaldehyde-releasing preservatives that present similar analytical challenges. Future studies could explore whether this strategy is suitable for characterizing the toxicokinetics of other compounds in this class.

## 5. Conclusions

This study established an analytical approach to characterize the toxicokinetics of a rapidly metabolized preservative using MDMH as a model compound. By developing a validated LC-MS/MS method for simultaneous analysis of the unstable parent compound (MDMH) and its stable metabolite (DMH), we overcame analytical limitations in assessing the safety of this compound. Our results demonstrate that MDMH exhibits rapid clearance and near-complete metabolic conversion to DMH, with prolonged systemic retention and complete bioavailability following transdermal administration. This extensive biotransformation suggests that toxicokinetic assessments based solely on the parent compound may not adequately reflect systemic exposure. As a stable metabolite, DMH can be utilized as a biomarker to quantify systemic exposure. Population toxicokinetic modeling enabled us to quantify processes that cannot be directly measured, demonstrating that 84% of the administered MDMH is absorbed and metabolized through the skin, with concomitant formation of formaldehyde.

The combination of metabolite analysis and population modeling resolves analytical limitations in characterizing unstable preservatives. This methodology applies to other rapidly metabolized compounds where parent molecules cannot be reliably quantified.

## Figures and Tables

**Table 1:** Composition (% *w*/*w*) of gel containing MDMH and DMH. The 2% MDMH and DMH concentration were selected to ensure adequate systemic exposure for toxicokinetic characterization (regulatory limit: 0.2% in the EU and Korea).

Compound	Ingredient	Composition (%)
MDMH	MDMH	2.0
	Carbomer 940	1.5
	0.2 N NaOH	5.0
	Water	91.5
DMH	DMH	2.0
	Carbomer 940	1.5
	0.2 N NaOH	5.0
	Water	91.5

**Table 2:** Intra- and inter-day accuracy and precision of MDMH and DMH in rat plasma.

QC Level	MDMH		DMH	
	Intra-Day (n = 5)	Inter-Day (n = 5)	Intra-Day (n = 5)	Inter-Day (n = 5)
HQC	102.8 ± 4.4	104.3 ± 3.9	110.8 ± 3.5	107.2 ± 5.2
MQC	105.0 ± 3.5	100.8 ± 3.6	113.0 ± 2.7	104.9 ± 3.1
LQC	109.6 ± 5.2	104.1 ± 3.9	112.4 ± 2.1	108.8 ± 2.8
LLOQ	111.4 ± 4.9	103.5 ± 9.1	108.2 ± 5.9	98.8 ± 12.0

**Table 3:** Stability of MDMH and DMH in rat plasma and processed samples under different conditions.

Stability	QC Level	MDMH (%)	DMH (%)
Short-term	High (n = 5)	99.7 ± 1.3	101.0 ± 1.9
	Low (n = 5)	102.6 ± 1.5	102.0 ± 1.4
Long-term	High (n = 5)	109.0 ± 3.9	112.0 ± 3.2
	Low (n = 5)	110.4 ± 1.1	106.2 ± 4.3
Freeze–thaw	High (n = 5)	94.0 ± 1.8	93.2 ± 5.8
	Low (n = 5)	90.1 ± 4.1	89.3 ± 3.8
Processed sample	High (n = 5)	112.0 ± 3.5	113.2 ± 1.5
	Low (n = 5)	98.9 ± 4.7	90.9 ± 6.9

**Table 4:** Non-compartmental toxicokinetic parameters of MDMH and DMH obtained following IV injection or TD application of MDMH or DMH at a dose of 50 mg/kg or DMH at a dose of 10 mg/kg in rats (n = 5, mean ± S.D.).

Parameter	MDMH IV (n = 5)		DMH IV (n = 5)
	MDMH	DMH	DMH
t_1/2_ (min) ^a^	0.4 ± 0.12	174.22 ± 12.16	181.27 ± 33.08
C_0_ (μg/mL)	77.91 ± 28.05	-	26.91 ± 18.30
C_max_ (μg/mL)	-	184.27 ± 106.69	-
T_max_ (min)	-	0.66 ± 0.31	-
AUC_last_ (μg·h/mL)	0.57 ± 0.19	247.68 ± 109.74	47.06 ± 29.87
AUC_infinity_ (μg·h/mL)	0.58 ± 0.18	303.66 ± 166.46	55 ± 36.79
CL (L/h/kg)	94.1 ± 34.84	-	0.24 ± 0.13
V_z_ (L/kg)	0.84 ± 0.04	-	1 ± 0.47
F_m_ to DMH (%)	-	97.59 ± 9.64	-

^a^ Half-life values are presented in minutes to maintain precision. Hour equivalents: MDMH (IV) = 0.4 ± 0.12 min (0.007 ± 0.002 h); DMH after MDMH (IV) = 174.22 ± 12.16 min (2.9 ± 0.2 h); DMH after DMH (IV) = 181.27 ± 33.08 min (3.02 ± 0.55 h).

**Table 5:** Non-compartmental toxicokinetic parameters of DMH obtained after transdermal application of gel containing 2% of MDMH (10.9 mg/kg) or 2% of DMH (10.7 mg/kg) in rats (n = 5, mean ± S.D.).

DMH Parameter	MDMH TD	DMH TD
	10.9 mg/kg (n = 5)	10.7 mg/kg (n = 5)
t_1/2_ (h)	3.47 ± 0.38	5.36 ± 2.18
C_max_ (μg/mL)	4.88 ± 1.81	6.46 ± 1.69
T_max_ (h)	4.67 ± 1.53	2 ± 1.73
AUC_last_ (μg·h/mL)	32.51 ± 8.35	40.51 ± 0.05
AUC_infinity_ (μg·h/mL)	38.46 ± 10.56	51.47 ± 5.53
Absolute bioavailability (%)	-	100.92 ± 17.99
F_m_ to DMH (%)	89.78 ± 24.15	-

**Table 6:** Population toxicokinetic parameter estimates for MDMH and DMH.

Parameter	Symbol	Unit	Mean (BSV)
Clearance for MDMH	CL_MDMH_	L/h	147.63 (3.96)
Clearance for DMH	CL_DMH_	L/h	0.19 (44.21)
Distribution clearance for DMH	CLD_DMH_	L/h	5.73 (71.8)
Central volume of distribution for MDMH	V_c,MDMH_	L	1.18 (14.19)
Central volume of distribution for DMH	V_c,DMH_	L	0.32 (146.11)
Peripheral volume of distribution for DMH	V_p,DMH_	L	0.43 (35.86)
Absorption rate constant for DMH into the central compartment from skin	k_a,DMH_	1/h	8.03 (38.33)
Absorption-metabolism rate constant for MDMH in skin	K_a,met_	1/h	0.31 (6.44)
Fraction of applied MDMH absorbed and metabolized in skin	F_a,met_	-	0.84 (16.28)

## Data Availability

The original contributions presented in this study are included in the article. Further inquiries can be directed to the corresponding authors.

## Abbreviations

MDMH	MDM hydantoin
DMH	DM hydantoin
IV	Intravenous
TD	Transdermal
POP-TK	Population toxicokinetic
